# Reframing the gender gap: masculine ideology as a predictor of academic behaviors

**DOI:** 10.3389/fpsyg.2026.1805150

**Published:** 2026-03-27

**Authors:** Hannah M. Baskin, Catherine M. Bain, Jenel T. Cavazos

**Affiliations:** Department of Psychology, University of Oklahoma, Norman, OK, United States

**Keywords:** academic outcomes, gender, imposter phenomenon, intelligence mindset, masculine ideology, study strategies

## Abstract

**Introduction:**

While gender differences in academic performance are well documented, traditional approaches have relied on gender identity as the primary explanatory variable, yet this framing may obscure more nuanced behavioral and ideological factors at play. This study examines how masculine ideology influences academic behaviors and outcomes among college students, with implications for predicting retention and academic success.

**Methods:**

Using SEM with a sample of 1,068 undergraduate students, we investigated how entity mindset, imposter phenomenon, and masculine ideology interact to affect study habits, help-seeking behaviors, and academic outcomes. Model comparisons were conducted to evaluate the relative explanatory power of masculine ideology vs. gender identity in predicting academic success.

**Results:**

Results indicate that students with traditional masculine ideology (TMI) who experienced imposter feelings were more likely to employ surface-level study approaches and exhibit maladaptive help-seeking behaviors compared to those with non-traditional masculine ideology (NTMI). Help-seeking behaviors significantly predicted GPA and course grades, while study habits showed no significant direct effect on academic outcomes. Model comparisons revealed that masculine ideology provided greater explanatory power for predicting academic success than gender identity alone, suggesting that behavioral patterns associated with traditional masculine norms influence academic performance across genders and represent a critical leverage point for retention efforts.

**Discussion:**

These findings support the development of targeted interventions that address masculine ideology’s influence on academic behaviors rather than traditional gender-based approaches, offering a more precise framework for identifying at risk students and improving retention rates. Because the effects of traditional masculine norms on academic behavior transcend gender identity, interventions framed around gender categories alone may be insufficiently targeted. Future work should explore how institutional environments either reinforce or attenuate traditional masculine norms, and whether targeted messaging around help seeking can shift outcomes for students regardless of gender identity.

## Introduction

For many college students, their first year is a time of significant adjustment as they acclimate to a new environment with unfamiliar academic expectations and must adapt to self-regulating their learning process, which is crucial for long-term achievement and graduation (Biwer et al., 2020; McDaniel et al., 2021; Moore et al., 2010; Respondek et al., 2019; Tuckman and Kennedy, 2011). Though most students experience this adjustment period, research indicates that men struggle to acclimate significantly more than women (Kaim and Romi, 2025). This represents a dramatic reversal from earlier in the twentieth century, when men were substantially more likely than women to earn a college degree (Goldin et al., 2006: Parker, 2015). Female bachelor’s degree attainment rose from 3.8% in 1940 to over 38% by the late 2010s (U.S. Census Bureau, 2023), and among adults aged 25–29 in 2022, 44% of women had completed a bachelor’s degree compared to only 35% of men (Quadlin et al., 2025). By 2024, women comprised approximately 60% of college enrollment, with academic outcome disparities emerging as early as middle school (National Student Clearinghouse Research Center, 2024). The consequences extend beyond individual outcomes, as men without college degrees face reduced competitiveness in the labor market and limited career advancement in an increasingly credential-focused economy (Goulas, 2024; Siegfried et al., 2007). Given the substantial investment and lifelong impact of college education, understanding these evolving gender dynamics has become increasingly critical for researchers and policymakers.

Traditional gender-based frameworks for understanding academic performance disparities have proven inadequate and potentially counterproductive. These approaches assume that gender identity itself drives academic differences, treating male and female students as homogeneous groups with distinct approaches to learning and failing to account for significant within-gender variation that helps explain why some male students succeed while others struggle (Kessels and Steinmayr, 2013; Petersen and Hyde, 2014). Gender-focused interventions have yielded mixed results; a systematic review found that 50% of interventions measuring social change in gender equality failed to demonstrate beneficial effects (Guthridge et al., 2022). For example, “boy-friendly” educational reforms (i.e., single-sex schooling, increased male teachers, and activity-based curricula) have ranged from ineffective to actively harmful (Bazinet, 2015). These interventions typically fail because they rely on biological determinism rather than addressing the social construction of masculinity (Bazinet, 2015). Consequently, researchers are shifting toward masculine ideology frameworks that examine beliefs about appropriate male behavior and attributes, such as effortless achievement and rejection of help-seeking behaviors, which operate independently of gender identity and may be more malleable targets for intervention than deeply held gender identities (Bui et al., 2023; Jackson and Dempster, 2009; Jugović, 2017).

### Masculine ideology and academic success

Masculine ideology represents a set of standards and expectations defining masculinity. In the United States, this is characterized by traditional masculine ideology (TMI) dimensions including achievement, emotional control, antifemininity, and homophobia (Levant and Powell, 2017; Pleck, 1995). This construct transcends gender identity, as both men and women can endorse traditional masculine beliefs, establishing it as a reliable measure of individual differences across genders (Borgogna and McDermott, 2022). In academic contexts, TMI encompasses beliefs about “acceptable” student behaviors, often reflecting broader social attitudes where academic behaviors like goal setting and help-seeking are perceived as feminine traits, leading students who endorse traditional masculine beliefs to suppress academically beneficial behaviors that conflict with these norms (Brown et al., 2021; Liang et al., 2019).

The theoretical foundation for masculine ideology has been substantially developed through frameworks such as the Gender Role Strain Paradigm (Pleck, 1981, 1995), resulting in validated instruments that assess masculinity standards, gender role conflict, and conformity to masculine norms (Levant and Richmond, 2008; Thompson et al., 1992). For example, research demonstrates that masculine ideology predicts academic outcomes beyond gender identity alone: students with higher endorsement of traditional masculine ideology achieve lower grades and GPAs, demonstrate reduced academic engagement, and show higher withdrawal rates compared to those with lower endorsement (Kessels and Steinmayr, 2013; Marrs, 2016; Schwab, 2018). Despite these theoretical contributions, masculine ideology remains underutilized in educational research, creating a problematic gap given evidence that masculine stereotypes emphasizing “effortless achievement” position academic effort and help-seeking as incompatible with masculine identity (Workman and Heyder, 2020). This framework offers advantages over traditional gender-based approaches by recognizing that beliefs about appropriate behavior drive academic choices, acknowledging within-gender variation in academic success, and suggesting intervention opportunities targeting malleable belief systems rather than fundamental identity aspects.

### Factors influencing academic success

#### Study strategies

Masculine ideology shapes how students approach learning, with certain strategies perceived as more or less aligned with masculine norms (Grabill et al., 2005; Jackson and Dempster, 2009). Research categorizes study strategies into deep approaches (e.g., concept mapping, elaborative rehearsal) that build meaningful connections, versus surface approaches (e.g., rote memorization, flashcard drilling) that meet minimal requirements (Brown and Murdolo, 2017; Lindblom-Ylänne et al., 2019; Ward, 2011). Individuals lower in TMI tend to employ deep approaches more frequently. In contrast, those higher in TMI are more likely to rely on surface approaches, reflecting how traditional masculine norms emphasizing effortless achievement align with surface strategies that promise faster results (Marrs, 2016; Marrs and Sigler, 2012; Noviana et al., 2020). Cultural messages framing sustained intellectual effort as “feminine” create barriers to deep learning, as the vulnerability and effort required conflict with masculine ideals of projecting confidence and natural ability (Chaffee et al., 2020; Lei and Lundberg, 2020).

Study strategy interventions show mixed results. For example, while Tuckman and Kennedy (2011) found that teaching learning strategies increased success among first-term college students, Hattie (2015) reported that strategy interventions in higher education show variable effectiveness depending on implementation context. One contributing factor is that students often persist with familiar approaches even after being introduced to more effective techniques, a tendency that reflects gender socialization patterns established in early childhood, where male students may receive messages that devalue visible academic effort (Hattie, 2015; Javornik and Klemenčič Mirazchiyski, 2023). Individuals low in masculine ideology tend to demonstrate higher intrinsic academic motivation and employ more effective study strategies than those high in masculine ideology.

#### Mindset and the imposter phenomenon

Traditional masculine ideologies promote effortless achievement, in which academic success must appear natural, emphasizing innate talent over visible effort, thereby aligning with entity-mindset beliefs about fixed intelligence (Elliott and Dweck, 1988; Jackson and Dempster, 2009; Kumar and Jagacinski, 2006). Mindset theory describes incremental (growth) and entity (fixed) orientations that shape how individuals conceptualize intellectual capabilities (Dweck and Leggett, 1988). Incremental mindsets challenge traditional masculine ideology by embracing vulnerability and effort-based growth rather than displays of natural ability, fostering task-involving goals focused on understanding rather than performance (Dweck, 1986). Entity mindsets align with masculine performance expectations by emphasizing fixed ability and avoiding displays of incompetence, with students defining competence through competitive comparisons that reflect masculine values of dominance (Dweck, 1986; Kumar and Jagacinski, 2006; Liu, 2021). Students with entity mindsets tend to demonstrate lower academic achievement and reduced persistence when faced with challenges, while those with incremental mindsets show greater resilience and improved academic outcomes (Dweck, 2006; Liu, 2021). The intersection of masculine ideology and entity mindset creates a self-reinforcing cycle in which students feel compelled to perform with effortless competence while internally doubting their abilities, thereby transforming normal academic challenges into threats to both intellectual and gender identity (Kumar and Jagacinski, 2006; Nicholls, 1984; Rogers et al., 2020; Leaper et al., 2019; Cokley et al., 2015; Vandello et al., 2008; Vescio et al., 2021).

The imposter phenomenon (IP) involves feelings of fraudulence despite objective success. Although prevalence does not differ by gender, there is significant variation in how it is experienced based on mindset and masculine ideology (Clance and Imes, 1978). Individuals with entity mindsets are particularly susceptible to imposter feelings as they perceive intellectual capabilities as unchangeable (Kenneally et al., 2023; Noskeau et al., 2021). Individuals high in traditional masculine ideology (TMI) are more likely to respond to imposter feelings by adopting ability-avoidant goals and prioritizing failure avoidance over learning opportunities (Noskeau et al., 2021; Fassl et al., 2020; Leaper et al., 2019). This orientation is associated with tendencies toward selective class participation, avoidance of challenging tasks, selection of less demanding coursework, and reluctance to seek academic support (Fried-Buchalter, 1997; Wimer and Levant, 2011). In contrast, individuals lower in traditional masculine ideology tend to respond with perfectionism and increased effort driven by societal expectations to prove their worth, often experiencing achievement anxiety and lower confidence partly due to societal perceptions of their competence (Brown et al., 2021; Fried-Buchalter, 1997; Jackson and Dempster, 2009; Kosakowska-Berezecka et al., 2017; Perander et al., 2020). Originally identified among high-achieving women who were unable to internalize their academic and professional successes (Clance and Imes, 1978), the impostor phenomenon has since been documented across diverse student populations regardless of gender or achievement level (Bravata et al., 2020; Ménard, 2023). Regardless of its manifestation, the impostor phenomenon is associated with diminished academic motivation and lower academic performance (Kumar and Jagacinski, 2006; Vaughn et al., 2020).

#### Help-seeking behaviors

Traditional masculine norms emphasize self-reliance and view help-seeking as weakness that invalidates masculinity, leading individuals higher in TMI to tend to avoid academic assistance even with poor performance feedback (Doebling and Kazerouni, 2021; Wimer and Levant, 2011). When students high in TMI do seek help, they tend to engage in executive help-seeking (asking others to solve problems for them) and rely on peer support for validation, often attributing difficulties to external factors like course structure to maintain an image of effortless achievement (Jackson and Dempster, 2009; Kessels and Heyder, 2020; Perander et al., 2020; Ryan and Pintrich, 1997). Individuals lower in TMI tend to demonstrate different patterns, often viewing help-seeking as a valuable tool rather than a threat, and more often using instrumental help-seeking that requests specific information while maintaining independence (Marrs, 2016; Ryan and Pintrich, 1997; Severiens and ten Dam, 2012; Wimer and Levant, 2011). This difference correlates with measurable outcomes. Individuals with lower endorsement of traditional masculine ideology achieve higher grades, better GPAs, and lower withdrawal rates than those who strongly endorse traditional masculine ideologies (Kessels and Steinmayr, 2013; Schwab, 2018). By contrast, those higher in traditional masculine ideology are less likely to utilize academic support services, suggesting that efforts to address achievement gaps may benefit from greater attention to how cultural norms shape learning approaches (D. Brown et al., 2021; Kuśnierz et al., 2020; Marrs, 2016; Taplin and Jegede, 2010).

### Current project

Although previous research has established relationships among mindset, the imposter phenomenon, study strategies, help-seeking, and masculine ideology, no comprehensive model integrating these variables has been developed. Current research is limited by small sample sizes, artificial laboratory settings, or a narrow focus on single academic outcomes (Javornik and Klemenčič Mirazchiyski, 2023). This study addresses these limitations by examining multiple real-world academic outcomes (GPA, course grades, academic retention) in a large sample of college students and systematically testing masculine ideology as a moderator of the relationships between psychological factors and academic behaviors across genders. This approach moves beyond simple gender comparisons to examine specific pathways through which endorsement of traditional masculine ideology affects academic success.

### Study description and hypotheses

The present study had three primary aims. First, we examined whether traditional masculine ideology moderates the relationship between the imposter phenomenon and academic help-seeking behaviors. Second, we aimed to test the interactive effects of entity mindset and masculine ideology on maladaptive study habits, particularly in the context of elevated imposter feelings. Third, we aimed to determine the relative contribution of help-seeking behaviors and study habits in predicting academic outcomes, and whether these pathways differ as a function of individuals’ endorsement of traditional masculine ideology.

Based on prior research, we hypothesized that individuals who endorse a stronger entity mindset would report higher levels of the imposter phenomenon. We further expected that masculine ideology would moderate the relationship between the imposter phenomenon and academic behaviors. Specifically, among individuals experiencing elevated imposter feelings, those who more strongly endorse traditional masculine ideology were expected to demonstrate more maladaptive study habits than those who endorse less traditional masculine ideology. In addition, endorsement of traditional masculine ideology was expected to predict fewer academic help-seeking behaviors, such as attending office hours and using tutoring services, than endorsement of non-traditional masculine ideology. The relationship between these psychological factors and academic outcomes was expected to be indirectly mediated by study habits and help-seeking behaviors, such that maladaptive study habits would negatively predict academic performance. In contrast, greater help-seeking would positively predict academic performance. These indirect effects were expected to be conditional on masculine ideology, with traditional masculine ideology strengthening the negative association between imposter phenomenon and help-seeking behaviors and amplifying the association between imposter phenomenon and maladaptive study habits. Consequently, poorer academic outcomes were anticipated for individuals who experienced higher levels of imposter phenomenon and more strongly endorsed traditional masculine ideology.

## Method

### Participants

A total of 1,382 students enrolled in an introductory psychology course at a large Midwestern university participated in this study in exchange for course research credit. Of the original sample, 314 participants were excluded for failing to respond correctly to embedded attention checks, resulting in a final sample of 1,068 participants. The sample comprised 74.9% female-identifying and 25.1% male-identifying individuals. Participants ranged in age from 18 to 31 years (M = 18.94, SD = 1.59), and the majority were first-year students (65.3%), with the remainder second-year or above. Most respondents identified as White or Caucasian (79.7%). Additional racial identities endorsed included Asian (9.7%), American Indian/Native American or Alaska Native (8.9%), Black or African American (7.9%), and Native Hawaiian or Other Pacific Islander (0.7%). A small proportion of participants selected “Other” (3.2%) or preferred not to report their race (1.6%). Percentages exceed 100% because participants could endorse more than one racial category.

### Measures

#### Help-seeking behaviors

The Psychology Help-Seeking Scale (PHSS) is a 36-item measure that uses an 8-point Likert-type scale from 1 (*most definitely false*) to 8 (*most definitely true*) to assess help-seeking behaviors (Pajares et al., 2004). The PHSS includes four subscales: Instrumental Help-Seeking (α = 0.88), Executive Help-Seeking (α = 0.94), Avoidance of Help-Seeking (reverse-scored; α = 0.92), and Perceived Benefits of Help-Seeking (α = 0.89). A sample item is included: “When I ask my psychology teacher for help, I prefer to be given hints or clues rather than the answer.” In addition to the PHSS, help-seeking was assessed using real-world behavioral measures that were collected during the semester of study participation. Higher subscale scores indicate greater use of that particular type of help-seeking. These measures include data on visits to psychology tutoring sessions, study reviews, and the instructor’s and graduate assistant’s office hours.

#### Masculinity

The Conformity to Masculine Norms Inventory-30 (CMNI-30; Levant et al., 2020) is a 30-item measure adapted from the original 94-item Conformity to Masculine Norms Inventory (CMNI; Mahalik et al., 2003), administered on a six-point Likert-type scale ranging from 1 (*strongly disagree*) to 6 (*strongly agree*). The CMNI-30 has 10 factors: Emotional Control, Winning, Playboy, Violence, Heterosexual Self-Presentation (*α* = 0.95), Pursuit of Status (α = 0.65), Primacy of Work (α = 0.82), Power Over Women (α = 0.81), Self-Reliance (α = 0.78), and Risk-Taking (α = 0.82). A sample item is: “I need to prioritize my work over other things.” Higher subscale scores indicate greater identification with that particular facet of masculine ideology.

#### Imposter phenomenon

The Clance-Imposter Phenomenon Scale (CIPS) measures perceived incompetence and the ability to perform well in academic settings. Using a 20-item scale (α = 0.92; Clance, 1985), participants rate their responses on a 5-point Likert-type scale ranging from 1 (*not true at all*) to 5 (*very true*). A sample item is included: “When I have succeeded at something and received recognition for my accomplishments, I have doubts that I can keep repeating that success.” Higher scale scores indicate greater feelings of imposter phenomenon.

#### Mindset

The Growth Mindset Scale (GMS) uses a three-item Likert-type scale ranging from 1 (strongly agree) to 6 (strongly disagree; α = 0.91) to measure the belief that intelligence is malleable (Dweck, 1986). A sample item is included: “You have a certain amount of intelligence, and you cannot really do much to change it.” Higher scale scores indicate greater identification with an incremental (growth) mindset.

#### Study strategies

The Revised Study Process Questionnaire-Two Factor (R-SPQ-2F) is a 20-item scale used to measure students’ deep and surface learning approaches (Biggs et al., 2001). The items are scored on a 5-point Likert-type scale ranging from 1 (*never or only rarely true*) to 5 (*always or almost always true*). This measure includes two factors: deep approach (α = 0.84) and surface approach (α = 0.80). Sample items include: “I find most new topics interesting and often spend extra time trying to obtain more information about them,” and “I aim to pass the course while doing as little work as possible.” Higher subscale scores indicate greater use of that study strategy type.

#### Academic outcomes

Academic outcome data was gathered at the University-level, and were assessed with three behavioral measures: (1) the participant’s final percentage grade in their introductory psychology course; (2) the participant’s overall GPA for the semester in which they participated in the study; and (3) the participant’s academic retention status, defined as whether the student reenrolled in the subsequent academic year following participation in the study.

## Procedure

Once IRB approval was received, participants were recruited via the psychology department’s experiment management system and then redirected to an online survey system to provide informed consent and complete the survey. Once all responses were collected, student response identification numbers were used to match responses with GPA, retention information, and the frequency of tutoring and office hours visits.

## Preliminary analysis

To evaluate the unique contribution of traditional masculine ideology beyond gender alone, we tested two alternative models. The first alternative model replaced traditional masculine ideology with gender as the moderating variable, allowing us to examine whether gender-based differences (i.e., being a man vs. a woman) account for variation in the relationships among the imposter phenomenon, study habits, help-seeking behaviors, and academic outcomes. The second alternative model excluded both gender and traditional masculine ideology, providing a baseline for assessing whether individual differences in masculine ideology offer explanatory value beyond general patterns that may exist regardless of gender or endorsement of traditional masculine ideology. These alternative models serve as a critical test of whether traditional masculine ideology provides unique insight into academic behaviors and outcomes beyond what can be explained by gender category alone.

Structural Equation Modeling (SEM) was conducted using R version 4.2.1, with *a priori* power analysis confirming that our sample of 1,068 participants exceeded the minimum requirements for detecting effects given the model complexity (Soper, 2025; Westland, 2010). Confirmatory factor analysis of the “Help-Seeking” latent variable, which combined self-report and behavioral measures, demonstrated good fit (RMSEA = 0.014, SRMR < 0.067), with all variables contributing significantly to the factor. Missing data were handled using Full Information Maximum Likelihood (FIML) in accordance with expert recommendations (Cham et al., 2017).

## Results

### Primary model

#### Estimation and specification

Models were estimated via robust maximum likelihood (MLR) in R, using the lavaan package. The model included observed variables (entity mindset, imposter phenomenon, GPA, final grade, and retention) and latent variables representing Masculine Ideology, Study Habits, and Help-Seeking. Missing data were handled using full information maximum likelihood (FIML). The Study Habits latent factor was assessed using the deep and surface learning approach subscales from the R-SPQ-2F. Factor loadings were significant for both indicators (deep: *β* = 1.000; surface: β = 2.010, SE = 0.447, *p* < 0.001). The Help-Seeking latent factor was indicated by four subscales from the PHSS (executive, instrumental, avoidance, and perceived benefits of help-seeking), as well as tutoring center use and office hours attendance. Because executive help-seeking served as the reference indicator (*β* = 1.000), the direction of all remaining loadings is interpreted relative to it: a positive loading indicates that the indicator moves in the same direction as executive help-seeking, whereas a negative loading indicates that the indicator moves in the opposite direction. Accordingly, higher scores on the Help-Seeking latent factor reflect a profile characterized by greater executive and avoidance help-seeking, and lower instrumental help-seeking, perceived benefits, tutoring use, and office hours attendance.

This directional pattern is theoretically coherent. Executive help-seeking, seeking answers directly from others rather than developing understanding independently, and avoidance of help-seeking both reflect disengagement from effortful, self-regulated learning. By contrast, instrumental help-seeking (seeking help to understand rather than just obtain answers) and perceived benefits of help-seeking are associated with more adaptive, autonomous engagement. Their negative loadings thus indicate that students scoring higher on the latent factor are less instrumentally oriented and perceive fewer benefits to seeking help. Similarly, negative loadings for tutoring frequency (*β* = −0.090, SE = 0.040, *p* = 0.024) and office hours attendance (*β* = −0.165, SE = 0.085, *p* = 0.052) indicate that students higher on the latent factor made less use of these resources, consistent with an avoidant or passively dependent help-seeking style. The remaining loadings were: instrumental help-seeking (*β* = −0.851, SE = 0.081, *p* < 0.001), perceived benefits (*β* = −0.719, SE = 0.284, *p* = 0.011), and avoidance (*β* = 0.987, SE = 0.365, *p* = 0.007).

Model fit was evaluated using multiple goodness-of-fit indices. Overall, the model demonstrated acceptable fit given its complexity: χ^2^(4148) = 12,178.71, p < 0.001; RMSEA = 0.043 (90% CI [0.042, 0.044]); robust RMSEA = 0.042; CFI = 0.829; robust CFI = 0.837; TLI = 0.823; robust TLI = 0.832; SRMR = 0.093. Although CFI and TLI values fall below conventional thresholds, these indices are known to be systematically deflated in complex, large-variable models and are sensitive to chi-square inflation in large samples (Shi et al., 2019). RMSEA values indicated good fit, consistent with recommendations for highly parameterized models (Vitoratou et al., 2022). With both RMSEA and SRMR reflecting acceptable fit and no theoretically justifiable basis for model modification, the overall fit profile is interpreted as sufficient for structural interpretation, consistent with Hu and Bentler's (1999) recommendation to evaluate fit indices conjointly rather than in isolation.

#### Mindset, imposter phenomenon, and antecedent relationships

An incremental (growth) mindset significantly predicted lower levels of the imposter phenomenon (*β* = −1.385, SE = 0.440, *p* = 0.002), supporting the hypothesis that individuals endorsing a stronger entity mindset would report higher levels of the imposter phenomenon. Mindset also directly predicted lower levels of masculine ideology (*β* = −0.045, SE = 0.014, *p* = 0.001). The imposter phenomenon was also a significant negative predictor of masculine ideology (*β* = −0.002, SE = 0.001, *p* = 0.004).

#### Moderation of study habits and help-seeking by masculine ideology

Imposter phenomenon was a significant predictor of study habits (*β* = 0.005, SE = 0.001, *p* < 0.001). Importantly, the interaction between imposter phenomenon and masculine ideology significantly predicted study habits (*β* = 0.610, SE = 0.242, *p* = 0.012), indicating a moderation effect. Among individuals experiencing higher levels of the imposter phenomenon, those higher in masculine ideology exhibited more maladaptive study patterns, characterized by greater reliance on surface approaches than on deep learning strategies. Entity mindset was not a significant direct predictor of study habits (*β* = −0.014, SE = 0.012, *p* = 0.243).

Imposter phenomenon was not a significant direct predictor of help-seeking behaviors (*β* = 0.004, SE = 0.004, *p* = 0.295). However, the interaction between imposter phenomenon and masculine ideology significantly predicted help-seeking (*β* = 0.610, SE = 0.242, *p* = 0.012), suggesting that masculine ideology fully moderated this relationship. For students experiencing imposter feelings, higher masculine ideology was associated with greater executive and avoidant help-seeking, lower instrumental help-seeking and perceived benefits, and reduced engagement with tutoring services and office hours. Mindset was a significant direct predictor of help-seeking behaviors (*β* = −0.149, SE = 0.034, *p* < 0.001), such that a stronger entity mindset was associated with more maladaptive help-seeking patterns.

#### Study habits, help-seeking, and academic outcomes

**Figure 1:**
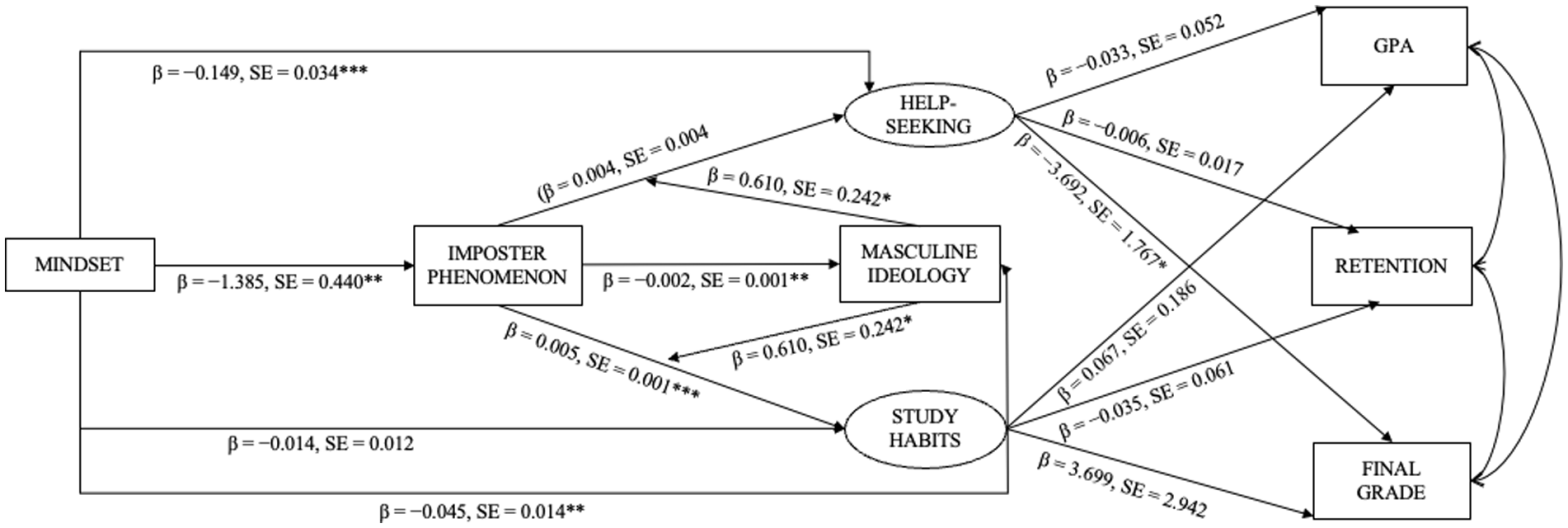
Help-seeking was measured using the PHSS, office hours, and tutoring center visits; study habits were measured using the RSPQ; * < 0.05, ** < 0.01, *** < 0.001.

Study habits were not significant predictors of GPA (*β* = 0.067, SE = 0.186, *p* = 0.719), final course grade (*β* = 3.699, SE = 2.942, *p* = 0.209), or retention (*β* = −0.035, SE = 0.061, *p* = 0.571). Help-seeking also did not significantly predict GPA (*β* = −0.033, SE = 0.052, *p* = 0.524) or retention (*β* = −0.006, SE = 0.017, *p* = 0.725). However, help-seeking was a significant predictor of final course grade (*β* = −3.692, SE = 1.767, *p* = 0.037), such that students exhibiting more avoidant and executive help-seeking patterns, as well as lower engagement with support resources, earned lower final grades. Final course grade (*β* = 0.024, SE = 0.005, *p* < 0.001) and retention (*β* = 0.378, SE = 0.131, *p* = 0.004) were both significant predictors of GPA. Final course grade was not significantly predicted by retention (*β* = 2.349, SE = 2.107, *p* = 0.265) (Figure 1).

#### Alternate models

**Figure 2:**
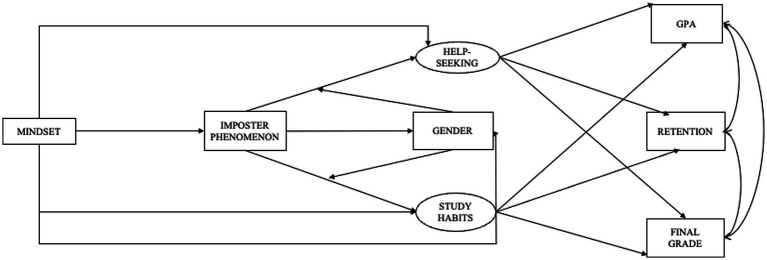
Alternative model. Help-seeking was measured using the PHSS, office hours, and tutoring center visits; study habits were measured using the RSPQ; specific path estimates were excluded due to the model’s lack of fit.

**Figure 3:**
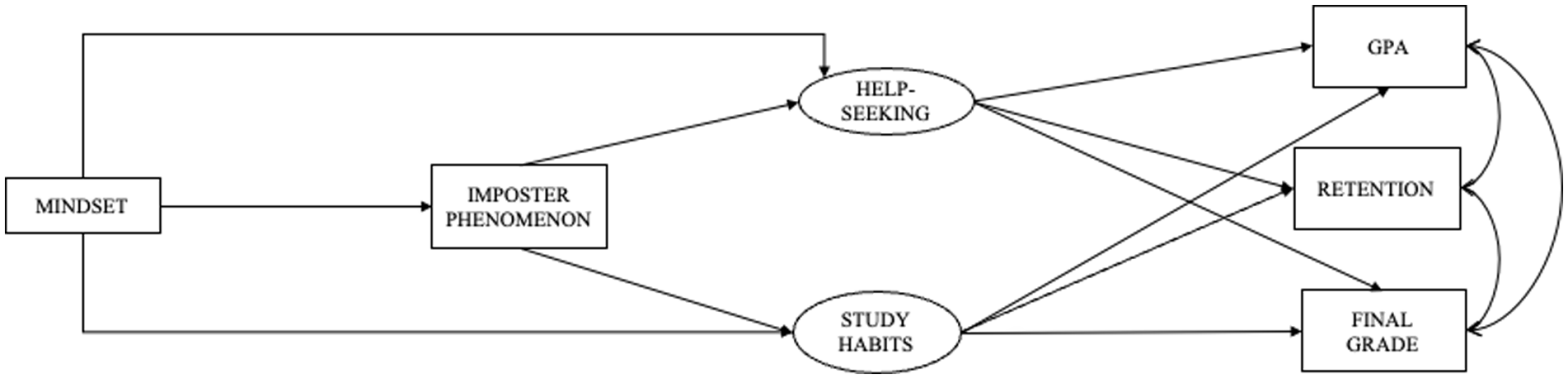
Model without moderation. Help-seeking was measured using the PHSS, office hours, and tutoring center visits; study habits were measured using the RSPQ; specific path estimates were excluded due to the model’s lack of fit.

To determine the relative importance of masculine ideology versus gender identity as moderators, an alternative model that substituted self-reported gender (binary male/female only, due to low response rates for other identities) was analyzed (Figure 2). This model demonstrated extremely poor fit (χ^2^ = 7498.130, df = 1926, *p* < 0.001, RMSEA = 0.053, TLI = 0.682, CFI = 0.711, SRMR = 0.123), indicating reduced predictive power compared to the masculine ideology model. Although the degrees of freedom differ across models due to changes in the number of estimated parameters, this is expected when comparing theoretically distinct models, and fit indices remain directly comparable because they adjust for model complexity (Kline, 2016). Finally, a model without gender or masculine ideology was analyzed to determine the relative importance of these variables (Figure 3). This model demonstrated an extremely poor fit (*χ*^2^ = 7660.288, df = 1,689, *p* < 0.001, RMSEA = 0.055, TLI = 0.680, CFI = 0.706, SRMR = 0.138) with non-significant pathways, confirming the critical role of masculine ideology in the model. Because both alternative models had extremely poor fit and therefore no predictive validity, we did not conduct further analyses using them.

## Discussion

The hypothesized model (Figure 1) illustrates predictive pathways alongside a good-to-moderate model fit. Significant individual paths suggest that the model captures several theoretically meaningful associations among the study variables, though numerous structural paths to GPA and retention were non-significant, indicating that the model reflects associations within a specific observational context rather than a comprehensive account of academic outcomes. These findings contribute to a theoretical framework for understanding the study variables and their interactions, while recognizing the limitations inherent in correlational, cross-sectional structural equation modeling.

### Entity mindset and the imposter phenomenon

An entity mindset significantly predicted the imposter phenomenon, supporting our first hypothesis that participants who viewed intelligence as fixed were more prone to perceived intellectual fraudulence than those who perceived intelligence as malleable, consistent with research showing that entity theorists adopt performance rather than mastery goals (Dweck, 1986; Kumar and Jagacinski, 2006). This finding extends beyond simple hypothesis confirmation: when intelligence is viewed as a fixed trait, academic setbacks are more likely to be interpreted as evidence of stable, uncontrollable inadequacy rather than as informational feedback receptive to effort and strategy, thereby sustaining the cognitive conditions that perpetuate impostor feelings over time (Ibrahim et al., 2023; Neureiter and Traut-Mattausch, 2016). While men and women experience entity mindset and imposter phenomenon at similar rates, they differ in coping mechanisms (Kenneally et al., 2023; Noskeau et al., 2021), a distinction that is consequential because entity mindset and the impostor phenomenon may create self-reinforcing cycles in which academic difficulty is reinterpreted as confirmation of inadequacy, further solidifying receptive avoidance-based coping across demographic groups (Ménard, 2023). This suggests academic support services should develop targeted interventions addressing distinct coping styles across demographic groups, with faculty training being particularly important since these patterns emerge as early as middle school (Marrs and Sigler, 2012).

### Imposter phenomenon as a predictor of help-seeking

While imposter phenomenon showed no direct relationship with help-seeking behaviors, masculine ideology significantly moderated this relationship. This pattern is theoretically significant: the absence of a direct effect of imposter phenomenon on help-seeking, combined with a significant moderation, suggests that masculine ideology functions as a necessary activating condition rather than simply an additive predictor. That is, impostor feelings alone do not reliably predict maladaptive help-seeking; rather, it is the intersection of those feelings with traditional masculine norms emphasizing self-reliance and effortless competence that produces avoidant and executive patterns of academic support-seeking. NTMI individuals demonstrated higher instrumental help-seeking and use of academic support services, whereas TMI individuals exhibited executive help-seeking and avoidance patterns, consistent with research on effortless achievement ideals that lead to maladaptive behaviors (Jackson and Dempster, 2009; Marrs, 2016; Severiens and ten Dam, 2012; Wimer and Levant, 2011). TMI individuals’ tendency to avoid situations that reveal incompetence, rather than engaging in learning, is associated with sustained imposter feelings and may be associated with poorer academic outcomes (Doebling and Kazerouni, 2021). These findings suggest that future experimental or intervention research might explore whether reframing academic support services as “advanced problem-solving sessions” or “expert consultations” might improve engagement among TMI students who resist traditional help-seeking due to concerns about self-reliance (Wimer and Levant, 2011). This reframing approach is consistent with emerging evidence on gender-sensitive interventions, which has found that repositioning help-seeking as an act of strategic self-improvement aligned with masculine values of mastery and competence, rather than as a sign of weakness, significantly improves service engagement among individuals who endorse traditional masculine norms (Sagar-Ouriaghli et al., 2023; Mokhwelepa and Sumbane, 2025).

### Masculine ideology as a predictor of study strategies

In the present model, the Study Habits latent factor represented a continuum of learning approaches, with higher scores indicating a more maladaptive pattern characterized by greater reliance on surface learning strategies relative to deep learning approaches. Consistent with prior research, individuals higher in traditional masculine ideology were more likely to exhibit this maladaptive study profile, particularly when experiencing elevated imposter feelings. These findings align with evidence that traditional masculine norms emphasizing efficiency, task completion, and effortless achievement discourage sustained cognitive engagement and the vulnerability required for deep learning (Asikainen et al., 2020; Lei and Lundberg, 2020). This pattern reflects a broader theoretical tension: deep learning, which requires sustained intellectual effort, tolerance for uncertainty, and willingness to revise understanding, is fundamentally incompatible with the effortless competence ideal embedded in traditional masculine norms (Vandello et al., 2008). Engaging visibly and effortfully with academic material may signal to TMI-endorsing students that they are struggling, which, when combined with elevated impostor feelings, creates conditions under which surface strategies become psychologically safer than mastery-oriented learning.

Although study habits did not directly predict academic outcomes in this predominantly first-year sample, the observed moderation effect suggests that masculine ideology shapes *how* students respond to academic threat. For individuals higher in traditional masculine ideology, imposter feelings were associated with greater reliance on surface learning strategies, whereas individuals lower in traditional masculine ideology demonstrated more adaptive learning patterns at similar levels of perceived inadequacy. This pattern supports prior work indicating that traditional masculine norms constrain students’ willingness to engage deeply with academic material, particularly in contexts that challenge competence or expose uncertainty (Marrs, 2016; Marrs and Sigler, 2012). Importantly, the present findings extend this prior work by demonstrating that the effect of masculine ideology on study habits is not a stable main effect but rather a conditional relationship that becomes pronounced specifically when impostor feelings are elevated, suggesting that the combination of these two psychological states constitutes a distinct risk profile for surface learning reliance that neither variable produces independently. These findings suggest that interventions targeting study strategies may be most effective when they explicitly address the belief systems that frame sustained academic effort as incompatible with competence or masculinity. Reframing deep learning strategies as tools for mastery, efficiency, and expertise, rather than as indicators of struggle, may reduce resistance among students who strongly endorse traditional masculine norms.

### The role of masculine ideology

Our findings suggest that masculine ideology, rather than binary gender identity, is more meaningfully associated with academic behavior patterns in the present sample. Both alternative models, one with gender as the moderator (Figure 2) and the model without moderators (Figure 3), showed substantially poorer fit, providing comparative support for the role of masculine ideology as a construct. However, it is important to note that several structural paths in the primary model, including the majority of paths to GPA and retention, were non-significant. These comparisons therefore support the relative theoretical utility of masculine ideology over gender as a modeling variable, not a claim that masculine ideology comprehensively determines academic outcomes. These findings suggest that assessing masculine ideology, rather than relying solely on binary gender, may offer a more theoretically precise basis for understanding students’ academic behavior patterns, and that future intervention research might consider attending to masculine ideology when designing outreach and support messaging for TMI individuals (Brown et al., 2021; Kessels and Heyder, 2020; Perander et al., 2020; Taplin and Jegede, 2010).

### Academic outcomes

Help-seeking behaviors were associated with academic performance in the present model, though associations were outcome-specific. Maladaptive help-seeking patterns, characterized by executive and avoidant strategies, lower perceived benefits of help-seeking, and reduced engagement with academic support services, were significantly associated with lower final course grades. In contrast, help-seeking behaviors were not significantly associated with semester GPA or academic retention. These findings are consistent with the interpretation that, in this observational sample, help-seeking patterns may be more closely linked to proximal, course-level performance than to broader indicators reflecting cumulative or longer-term academic functioning, though causal conclusions cannot be drawn from correlational data. This differentiation between proximal and distal academic outcomes is consistent with theoretical accounts of how behavioral patterns generate their effects: avoidance of office hours, reduced tutoring use, and reliance on executive strategies represent course-level behavioral decisions whose consequences are captured most directly in the grade earned in the course where those decisions occur (Ménard, 2023). Their absence from GPA and retention predictions likely reflects the multi-semester aggregation of those measures rather than a lack of meaningful effect.

Study habits did not significantly predict GPA, final course grade, or retention. This null finding may reflect the predominantly first-year composition of the sample, as students early in their academic careers may not yet have developed stable or effective study routines that reliably translate into performance differences across outcomes. Additionally, academic adjustment during the transition to college may attenuate the relationship between learning strategies and first-semester grades, consistent with prior research on early college adaptation (Moore et al., 2010; Respondek et al., 2019).

Although help-seeking did not directly predict GPA or retention, final course grade and retention both significantly predicted GPA, underscoring the interrelated nature of academic outcomes. Taken together, these findings suggest that help-seeking behaviors may warrant attention as a potential point of focus in future intervention research, given their observed association with course-level performance and the established link between course grades, GPA accumulation, and persistence in the present model. Whether targeted support for adaptive help-seeking would produce improvements in these outcomes remains an empirical question that longitudinal or experimental designs would be needed to address.

### Limitations and future directions

The predominantly first-year sample limits the validity of GPA as an outcome, as it reflects only a single semester of academic performance, which may have attenuated model fit and effect sizes. In addition, academic performance was indexed using grades from a single introductory psychology course, limiting generalizability to other disciplines and instructional contexts; future research should incorporate multi-course or cumulative GPA measures to better capture overall academic achievement. Measurement tools may not have fully differentiated between theoretically distinct constructs, suggesting the need for more parsimonious and discriminant instruments. Retention analyses were also restricted to subsequent-year enrollment rather than longer-term persistence to graduation. Finally, help-seeking behavior was operationalized solely through introductory psychology tutoring and office-hour usage, potentially overlooking help-seeking across other coursework (e.g., biology, chemistry, or history), where demands and support structures may differ.

The disproportionate representation of female participants may have reduced the observed association with masculine ideology, underscoring the need for larger, more gender-balanced samples. Future work should also examine cultural and social factors that contribute to women’s endorsement of traditional masculine ideology. Reliance on self-report measures introduces the possibility of response bias. Moreover, mindset may operate as a context-dependent construct rather than a stable trait, with students potentially adopting different mindsets across academic domains (e.g., major-related versus less intrinsically valued courses; Cypryańska and Nezlek, 2024). Future research should investigate how masculine ideology interacts with race, socioeconomic status, and first-generation status to inform targeted student support programming. Additionally, scholars should develop and evaluate intervention strategies that incorporate masculine ideology as a moderating variable and move beyond binary gender comparisons to elucidate the specific mechanisms through which traditional masculine ideology influences academic success (Dinsmore et al., 2023; Javornik and Klemenčič Mirazchiyski, 2023; Parnes et al., 2020).

### Recommendations for practice

Because this study was conducted with a predominantly first-year, female, and White sample drawn from a single introductory psychology course, the following recommendations are necessarily context-specific and should be interpreted as applicable primarily to comparable introductory educational settings pending replication in more diverse samples. With that caveat in mind, the present findings identify preliminary intervention strategies targeting the psychological factors and academic behaviors associated with student retention in this context. For example, growth mindset centered workshops might explicitly address how the combination of entity mindset and traditional masculine norms creates barriers to persistence, emphasizing that intellectual growth requires effort and help-seeking without threatening competence (Dweck, 2006). Given our finding that TMI predicts reduced help-seeking behaviors, interventions must directly challenge the masculine norm of self-reliance that prevents students from accessing critical academic support resources. Peer-led support programs could normalize help-seeking within same-identity groups, as students who endorse TMI may be more receptive to peer support than to formal institutional interventions (Kessels and Heyder, 2020). This approach is particularly important, given that our mediation analyses demonstrate that help-seeking behaviors are a key pathway through which the imposter phenomenon influences retention-related outcomes, such as GPA and course completion.

In introductory course settings like the one examined here, study strategy workshops might reframe deep learning strategies to emphasize mastery and competence development rather than vulnerability or struggle, directly addressing the finding that TMI moderates the relationship between imposter phenomenon and surface learning approaches. Explicitly naming and challenging the masculine norm that equates academic effort with low inherent ability, and that frames asking for academic support as an admission of intellectual inadequacy, may be particularly salient for TMI-endorsing students, who according to the present moderation findings are most likely to respond to imposter-related threat by retreating to surface-level, low-effort academic engagement. Because surface learning strategies negatively predict academic performance, a primary predictor of attrition, interventions that reduce surface learning adoption among high-TMI students may interrupt the pathway from psychological distress to academic failure and eventual departure. Faculty development programs in introductory courses should equip instructors to use language that avoids reinforcing entity-mindset beliefs, particularly when providing feedback or discussing academic challenges, as our findings suggest that entity mindset is a foundational factor that heightens imposter phenomenon and subsequently influences the very behaviors that determine whether students persist or leave. Instructor awareness of this dynamic may enable earlier, more appropriately framed outreach to students whose withdrawal reflects psychological barriers rather than motivational deficits. These recommendations are most directly applicable to instructors and support staff working with first-year students in gateway course contexts.

Within introductory course settings, early identification systems should screen for the constellation of entity mindset, imposter phenomenon, and TMI endorsement, as our moderated mediation model demonstrates these factors interact to predict retention-critical outcomes. Students identified as high-risk based on this psychological profile could receive targeted outreach that emphasizes available support resources and reframes help-seeking as a strategic behavior rather than an admission of inadequacy. These recommendations align with APA educational equity goals, emphasizing inclusive environments that address systemic barriers by examining socialized beliefs rather than inherent characteristics (APA Equity, Diversity, and Inclusion Framework, 2021). Critically, our alternative model comparisons revealed that TMI provides explanatory value beyond gender alone, suggesting that interventions focusing on belief systems rather than demographic categories create more equitable environments that support all students, regardless of gender identity, in developing the adaptive academic behaviors necessary for retention. However, because this study was conducted with a predominantly White, female, first-year sample at a single institution, replication across more racially, gender-, and institutionally diverse samples, and across multiple course contexts, is necessary before these recommendations can be adopted more broadly. The psychologically informed intervention strategies outlined here are best understood as preliminary and context-bound, representing a starting point for addressing educational equity challenges and attrition in first-year introductory course settings.

## Data Availability

The raw data supporting the conclusions of this article will be made available by the authors, without undue reservation.
